# Changing Cause of Death Profile in Morocco: The Impact of Child-survival Programmes

**Published:** 2007-06

**Authors:** Michel Garenne, Nada Darkaoui, Mhamed Braikat, Mustapha Azelmat

**Affiliations:** 1 Institut de Recherche pour le Développement and Institut Pasteur, Unité d’Epidémiologie des Maladies Emergentes, 25-28 rue du Docteur Roux, 75724 Paris Cedex 15, France; 2 Ministère de la Santé, Rabat, Morocco

**Keywords:** Causes of death, Child mortality, Child survival, Diarrhoea, Evaluation studies, Impact studies, Infant mortality, Interventions, Nutrition disorders, Respiratory tract infections, Vaccination, Morocco

## Abstract

This study was carried out to evaluate the trends in cause-specific mortality and the impact of child-survival programmes in Morocco. Two national surveys on causes and circumstances of child deaths were conducted in Morocco in 1988 and 1998 (ECCD-1 and ECCD-2 respectively). These surveys were based on a representative sample of deaths of children aged less than five years (432 and 866 respectively). Causes of death were assessed by verbal autopsy and were validated on a subsample of 94 cases. Data on causes of deaths were matched with death rates from demographic surveys (Enquête Nationale Démographique à Passages Répétés and Demographic and Health Survey) to compute cause-specific death rates. Morocco underwent a dramatic mortality decline since independence, and the decline in mortality among children aged less than five years was particularly rapid over the 1988-1997 period, at an average rate of −6% a year, and faster for children (aged 1-4 year(s)) than for infants. The decline in mortality varied markedly by causes of death and was most pronounced for causes due to vaccine-preventable diseases, such as neonatal tetanus, measles, whooping cough, tuberculosis, for diarrhoeal diseases and malnutrition, and for selected infectious diseases. However, mortality due to acute lower respiratory infection (ALRI) outside the neonatal period did not change significantly as was the case for some neonatal conditions (birth trauma and prematurity) and for accidents. The decline in cause-specific mortality could be attributed to the success of public-health programmes: the Expanded Programme on Immunization, the management of diarrhoeal diseases and malnutrition, and the use of antibiotics for selected infectious diseases. It is likely that improvements in living conditions, child-feeding practices, hygiene, and sanitation also contributed to the decline in mortality, although these could not explain the magnitude of the changes for target diseases. In contrast, the ALRI programme, which started after 1997, could not have any effect yet, and conditions of delivery and care of the newborn improved only marginally over the study period.

## INTRODUCTION

Infant and child mortality, measured by rate of deaths of children aged less than five years, underwent a dramatic decline in most countries in the world since 1950 (1). This decline in mortality is usually attributed to increasing income, improvements in living standards and nutrition, and progress in preventive and curative medicine and to national health programmes. Health programmes were limited in scope and coverage in the 1950s and 1960s.

Following the Alma Ata declaration in 1978, a new impulse was given, and the emphasis was shifted to the ‘community approach’ and to ‘primary health care’, with a focus on rural areas in developing countries. A series of large-scale programmes were launched in the 1980s and 1990s aimed at reducing child mortality. In particular, the so-called ‘Child Survival Programs’, sponsored by United States Agency for International Development and international organizations, launched around 1985, were very influential in numerous countries. These were based on a ‘selective primary health care approach’ and targeted a short list of diseases. They aimed at reducing all-causes mortality through cause-specific actions (2–5).

In typical Child Survival Programs, the main focus was on childhood diseases, and the main actions promoted were: comprehensive vaccinations to prevent some leading causes of death, such as measles, whooping cough, tetanus, diphtheria, poliomyelitis, and tuberculosis, the promotion of oral rehydration therapy (ORT) at the community level to prevent deaths from acute watery diarrhoea, and nutritional programmes, particularly supplementation of vitamin A. These programmes implied actions at the community level (training of mothers to use ORT, mass-vaccination campaigns, distribution of vitamin A, etc.).

Since 1995, a new wave of health programmes emerged, with emphasis on ‘Integrated Management of Childhood Illnesses’ within the health system. For communicable diseases, the main focus was on the diagnosis and treatment of acute lower respiratory infections (ALRIs) and other childhood infections, and in particular the proper use of antibiotics. In addition, more efforts were also devoted to maternal health: maternal nutrition, birth-preparedness, safe delivery, postpartum care, managing complications of pregnancy and delivery, spacing or birth intervals, some of which having an effect on neonatal mortality (USAID website). The effects of these programmes could be monitored by analyzing the mortality trends in population-based surveys, such as demographic and health surveys (DHSs). However, all these actions are expected to have a differential effect on cause-specific mortality, and only few programme evaluations have focused on cause-specific impacts, primarily due to lack of data on causes of death in developing countries (6,7).

Morocco is a good example of such health programmes over the past 25 years. The National Program on Immunization started in 1981 (PEV) and was reinforced in 1987 (PNI). It followed a comprehensive strategy of systematic vaccinations in health posts, of mobile teams going four times a year to rural areas, and on national vaccination days conducted each year. Vaccinations were provided, free of charge, to the whole population. The vaccination coverage increased dramatically over the period, from low values in 1982 to 50% in 1987 and 87% in 1997 (8,9).

A programme of combating diarrhoeal diseases (PLMD) was initiated in 1979, took off within a few years, and was further extended in 1990. In the first phase, the emphasis was on the use of ORT in the case of mild-to-moderate diarrhoea. In the second phase, more effort was devoted to train medical personnel to use ORT in clinics. As a result, the proportion of diarrhoea episodes treated with ORT increased from 14% in 1992 to 30% in 1997 and continued to increase ever since (10–13). This programme was conducted jointly with a concerted effort to improve basic hygiene.

The nutrition programme has been running for many years and included the promotion of exclusive breastfeeding for 4-5 months, child growth monitoring, prevention of vitamin D, vitamin A and iron deficiencies, and by improving the detection and treatment of severe malnutrition. Since 1998, vitamin A is distributed at the time of vaccination.

Several vertical programmes, such as a tuberculosis programme, a malaria programme, and a goitre programme, were also implemented, and specific actions taken against schistosomiasis and leprosy. The ALRI programme was set up only in 1997. Since 1998, many vertical programmes were integrated into the integrated management strategy (14).

On the maternal and delivery side, women were asked to make three visits to antenatal clinics during their pregnancy. Tetanus toxoid injections and iron supplementation were provided. However, the coverage of antenatal care remained limited, and deliveries often occurred at home, primarily because a large proportion of the population lives in remote areas, far from the nearest clinic, and lack safe transportation for pregnant women. In addition, the family-planning programme has been a major success in Morocco since its onset in 1965 (15,16).

The aim of this study was to document the changing profile in cause-specific mortality in Morocco over a nine-year period and to provide evidence for assessing the impact of child-survival programmes conducted in the 1980s and 1990s. This was done by survey methods, since the routine health information system remained weak over the period and took off only recently.

## MATERIALS AND METHODS

The method used for evaluating the impact of child-survival programmes focuses on changing cause-specific mortality rates. This evaluation requires mortality estimates and causes-of-death profiles.

Two surveys on causes and circumstances of child deaths were conducted in Morocco (17,18). The first survey—ECCD-1 (Enquête sur les Causes et Circonstances de Décès)—was conducted in 1988-1989 on a representative sample of deaths of children aged less than five years. The sample of deaths was drawn from a demographic multiround survey conducted in 1987-1988—the ENDPR (Enquête Nationale Démographique à Passages Répétés) (19). This demographic survey was conducted over a two-year period, with rounds every six months, and based on a fairly large representative sample of the population of Morocco (30,000 households). The sampling scheme was a random-stratified sample based on the master sample of the Statistics Division of the Ministry of Economic Affairs. All deaths of children under the age of five years that occurred between the third and the fifth round (deaths between October 1987 and October 1988) were selected for the enquiry on causes of death. The second survey—ECCD-2—was conducted in 1997-1998 on a sample of deaths recorded by the PapChild Survey (12). The PapChild Survey, a retrospective survey, was also based on a large representative sample of the population (45,000 households), which focused on births that occurred during the five preceding years (1993-1997). All deaths in this sample were selected for the ECCD-2. This second sample is, therefore, somewhat biased compared to that of the ECCD-1, since it does not include all deaths that occurred during the study period.

A verbal autopsy questionnaire was developed for Morocco in 1988 on the model of that developed in Senegal (20). The draft questionnaire was tested on some 30 cases in Rabat city and nearby villages in 1988, and the investigators adapted to best fit the local situation for the ECCD survey. The questionnaire included an open space for recording the history of the disease or accident that led to death, a comprehensive section of 18 pages on signs and symptoms, a section on socioeconomic circumstances of the case, and a section on care and treatments received. The questionnaire used in the second survey was virtually identical to that of the first survey, with only some minor changes.

The verbal autopsy surveys were conducted after the identification of deaths in the demographic surveys. In the first case, the delay between death and interview was eight months on average, whereas it was 41 months on average in the second survey, because of the inclusion of deaths that occurred several years before. The surveys were conducted by trained paramedics, who underwent a two-week training workshop. Two independent physicians reviewed the filled questionnaires, and in the case of disagreement, a third person reviewed until a final diagnosis was made. In the cases of further disagreement, the final diagnosis was coded as ‘unknown’.

Validation of the verbal autopsy technique was done by comparing verbal autopsy diagnosis with hospital diagnosis for those deaths that occurred in a hospital. Investigators of the verbal autopsies were blind to hospital diagnosis, and conversely persons who recorded the hospital diagnosis ignored the findings of verbal autopsy. In the first survey (ECCD-1), 57 deaths were matched with the hospital registers. Of these, 50 causes of death were identical: neonatal tetanus (17), birth trauma (7), neonatal infection (6), diarrhoea (6), pneumonia (2), neonatal jaundice (1), meningitis (3), septicaemia (1), congenital defect (2), epilepsy (1), prematurity (1), peritonitis (1), and accidents (2). Cases of discrepancies could come either from errors in verbal autopsy diagnosis (3 cases), or from inaccurate hospital records, typically in the cases where the child was brought to the hospital at the time of death or when diagnosis was incomplete in the hospital records (4 cases). In the second survey (ECCD-2), 37 deaths were matched with the hospital registers. Of these, 36 verbal autopsy diagnoses were identical to medical certificates: prematurity (8), birth trauma (7), neonatal infection (4), pneumonia (5), diarrhoea (3), meningitis (1), and congenital defect (8). The high degree of agreement between verbal autopsy diagnosis and hospital diagnosis in Morocco matched results from other validation studies on children aged less than five years conducted in the same way, such as that in South Africa (21).

Death rates were estimated from the demographic sample surveys for the periods covered by the verbal autopsy surveys: in the first case from the ENDPR, and in the second case from the DHS 2003-2004. The distribution of deaths by cause was used for computing rates of cause-specific mortality for three age-groups: neonatal (<28 days), postneonatal (28 days-11 months), and childhood (12-59 months). In the second survey, the bias in the sample was corrected by applying weights to deaths above age one year, year by year, to make the two samples comparable. More details are available in the survey reports (17,18).

Testing the mortality decline was done by comparing rates of cause-specific mortality between the two surveys and by applying standard t-test for differences between rates.

## RESULTS

### Mortality decline in Morocco

Mortality underwent a dramatic decline since independence in 1956. This decline in mortality could be reconstructed from the world fertility surveys (WFSs) and DHSs (11,13,22–24). Mortality of children aged less than five years was estimated at 234 per 1,000 in 1955-1959 (WFS) and at 47 per 1,000 in 1999-2003 (DHS 2003-2004). Data indicated a steady decline, although the speed was somewhat slower between 1956 and 1975 and somewhat faster after 1985 (Fig.). Mortality of children aged less than five years was estimated at 103 per 1,000 at the ECCD-1 survey (data from ENDPR for 1987-1988) and at 59 per 1,000 at the ECCD-2 survey (data from the DHS 2003-2004 for the 5-9 years before, i.e. 1994-1998). The decline in mortality between the two surveys was already rapid for neonatal deaths (-4.8% per year), very rapid for postneonatal deaths (−6.4% per year), and extremely rapid for children deaths (-9.9% per year). These data were used for estimating the decline in cause-specific mortality (Table 1).

**Fig.**
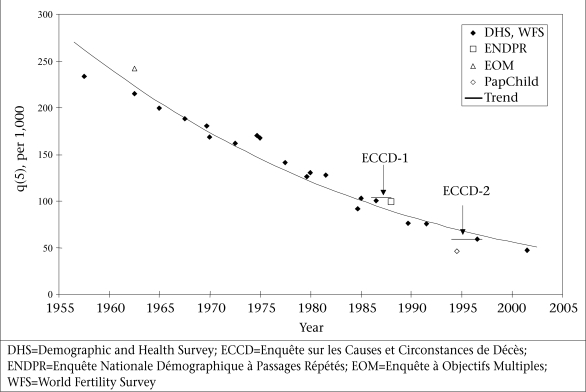
Trends in mortality of children aged less than five years, Morocco, 1956-2003

**Table 1:** Decline in mortality between the two surveys, Morocco

Source	Mean date	Death rates (per 1,000)				
		Neonatal (<28 days)	Postneonatal (28 days-11 months)	Infants (0-11 month(s))	Childhood (12-59 months)	Children (<5 years)
ENDPR	1987.5	45.6	31.6	75.7	30.7	104.1
DHS	1996.5	29.6	17.8	47.4	12.6	59.4
Mortality decline (%)		35.0	43.6	37.4	58.9	42.9
Mean annual decline (%)		−4.8	−6.4	−5.2	−9.9	−6.2

DHS=Demographic and Health Survey 2003-2004, data from 5-9 years before the survey (1994-1998) ENDPR=Enquête Nationale Démographique à Passages Répétés, 1987-1988

### Cause of death profiles

The decline in mortality between the two surveys was not homogenous by causes of death. For neonates, three causes of death underwent a statistically significant mortality decline: neonatal tetanus (-95%), pneumonia of the newborn (-57%), and low birth-weight (-57%), all declining faster than the others. Mortality from other causes also declined, with the exception of birth trauma, but the differences were not statistically significant (Table 2).

**Table 2:** Decline in neonatal mortality by cause between two ECCD surveys, Morocco, 1987-1996

Cause of death	ECCD-1			ECCD-2			Comparison 2/1	
	No. of deaths	%	Rate per 1,000	No. of deaths	%	Rate per 1,000	Decline (%)	p value
Birth trauma	26	20.3	9.26	136	33.7	9.99	−7.9	0.722
Premature	26	20.3	9.26	125	31.0	9.18	0.8	0.970
Low birth-weight	10	7.8	3.56	21	5.2	1.54	56.7	0.029∗
Tetanus	28	21.9	9.97	7	1.7	0.51	94.8	0.000∗
ALRI	12	9.4	4.27	25	6.2	1.84	57.0	0.016∗
Congenital defect	4	3.1	1.42	7	1.7	0.51	63.9	0.104
Diarrhoea, Acute	3	2.3	1.07	8	2.0	0.59	45.0	0.377
Other and unknown	19	14.8	6.76	74	18.4	5.44	19.6	0.395
Total	128	100.0	45.6	403	100.0	29.6	35.0	0.000

∗Signifcant (p<0.05); ALRI=Acute lower respiratory infection; ECCD=Enquête sur les Causes et Cir-constances de Décès

Postneonatal mortality declined for most causes and was significant for seven of them: diarrhoeal diseases (acute diarrhoea, chronic diarrhoea, dysentery being only borderline), tuberculosis, selected infectious diseases (meningitis, laryngitis, septicaemia), and malnutrition. Mortality from accidents, congenital defect, and other and unknown changed little, and the differences were not significant. For some critical diseases, such as measles, whooping cough, typhoid, the number of deaths was already small in the first sample, so that no conclusions could be made when analyzed disease by disease. More importantly, ALRI-related mortality did not decline significantly over the period, a striking difference from the other causes (Table 3).

**Table 3:** Decline in postneonatal mortality by cause between the two ECCD surveys, Morocco, 1987-1996

Cause of death	ECCD-1			ECCD-2			Comparison 2/1	
	No of deaths	%	Rate per 1,000	No of deaths	%	Rate per 1,000	Decline (%)	p value
ALRI	21	14.1	4.45	102	29.0	5.16	−15.9	0.538
Diarrhoea, Acute	33	22.1	6.99	73	20.7	3.69	47.2	0.002∗
Diarrhoea, Chronic	17	11.4	3.60	24	6.8	1.21	66.3	0.001∗
Dysentery	3	2.0	0.64	2	0.6	0.10	84.1	0.044
Total, diarrhoea							55.4	0.000∗
Tuberculosis	3	2.0	0.64	2	0.6	0.10	84.1	0.044∗
Malnutrition	35	23.5	7.42	64	18.2	3.24	56.4	0.001∗
Congenital defect	6	4.0	1.27	14	4.0	0.71	44.3	0.230
Hepatitis	1	0.7	0.21	7	2.0	0.35	−67.0	0.631
Typhoid	3	2.0	0.64	0	0.0	0.00	100.0	0.083
Whooping cough	1	0.7	0.21	5	1.4	0.25	−19.3	0.872
Laryngitis	5	3.4	1.06	0	0.0	0.00	100.0	0.025∗
Meningitis	9	6.0	1.91	9	2.6	0.46	76.1	0.002∗
Measles	2	1.3	0.42	1	0.3	0.05	88.1	0.083
Septicaemia	10	6.7	2.12	2	0.6	0.10	95.2	0.003∗
Accident	2	1.3	0.42	6	1.7	0.30	28.4	0.682
Other and unknown	33	22.1	6.99	87	27.6	4.91	29.9	0.167
Total	149	100.0	31.6	352	100.0	17.8	43.6	0.000∗

∗Signifcant (p<0.05); ALRI=Acute lower respiratory infection; ECCD=Enquête sur les Causes et Circonstances de Décès

Child mortality revealed a similar pattern, with an even faster decline. Causes of death that showed a significant decline were basically the same as for postneonatal deaths: diarrhoeal diseases (in this case acute diarrhoea and dysentery), malnutrition, tuberculosis, and selected infectious diseases (in this case typhoid, meningitis, and septicaemia). In this age-group again, mortality due to accidents and other and unknown causes did not change significantly, nor did ALRI-related mortality (Table 4).

**Table 4:** Decline in childhood mortality by cause between the two ECCD surveys, Morocco, 1987-1996

Cause of death	ECCD-1			ECCD-2			Comparison 2/1	
	No of deaths	%	Rate per 1,000	No of deaths	%	Rate per 1,000	Decline (%)	p value
ALRI	9	8.6	2.63	26	20.4	2.57	2.2	0.954
Diarrhoea, Acute	18	17.1	5.26	17	12.8	1.62	69.3	0.000∗
Diarrhoea, Chronic	11	10.5	3.21	19	16.7	2.10	34.6	0.263
Dysentery	9	8.6	2.63	2	2.4	0.30	88.4	0.006∗
Total, diarrhoea							63.8	0.000∗
Typhoid	5	4.8	1.46	1	1.0	0.13	91.2	0.027∗
Tuberculosis	4	3.8	1.17	2	1.8	0.22	81.0	0.055∗
Malnutrition	27	25.7	7.89	23	17.8	2.25	71.5	0.000∗
Congenital defect	3	2.9	0.88	5	4.0	0.50	42.8	0.444
Hepatitis	5	4.8	1.46	2	2.4	0.30	79.1	0.061
Nephritis	1	1.0	0.29	4	3.0	0.37	−27.3	0.829
Whooping cough	2	1.9	0.58	1	0.7	0.09	84.1	0.234
Laryngitis	1	1.0	0.29	0	0.0	0.00	100.0	0.317
Meningitis	6	5.7	1.75	2	1.5	0.19	89.4	0.006∗
Measles	3	2.9	0.88	2	1.5	0.19	78.8	0.089
Septicaemia	9	8.6	2.63	1	0.7	0.09	96.5	0.004∗
Accident	6	5.7	1.17	7	14.2	1.21	−2.2	0.948
Other and unknown	13	12.4	1.75	17	15.3	1.79	49.2	0.968
Total	105	100.0	30.7	112	100.0	12.6	58.9	0.000∗

∗Significant (p<0.05); ALRI=Acute lower respiratory infection; ECCD=Enquête sur les Causes et Circonstances de Décès

### Comparison with medical statistics

Registration of deaths is very limited in Morocco —about 20% according to the International Institute for Vital Registration and Statistics—and no comparison of death rates could be made with vital statistics. However, statistics on medical causes of death could be used for comparing the changing cause-of-death profiles. Data on causes of death recorded by the Ministry of Health (Bureau d’Hygiène) are notoriously deficient for children, and only 15% of causes of death of children were recorded at that time. However, despite the very low coverage, these data based on medical diagnosis allowed some of the survey findings to be confirmed. For neonates, the decline in the number of tetanus cases was of similar magnitude (92% in national causes of death statistics versus 95% in the ECCD surveys) and, likewise, for neonatal diarrhoea (67% vs 54%), for neonatal pneumonia (66% vs 57%), for prematurity (9% against 1% in the surveys), birth trauma (13% against 8% in the surveys), and for the total of all causes combined (35% in both cases). For postneonatal and childhood deaths, the agreement between the two sources was also good for several causes. The decline in the number of deaths was similar for all causes combined (44% in both cases) and for selected causes: diarrhoea (74% and 55% respectively in the two age-groups), tuberculosis (58% vs 84%), and measles (96% vs 88%). However, other causes appeared different and seemed poorly assessed by the ECCD surveys: typhoid (36%), meningitis (9%), septicaemia (16%), whooping cough (54%), and pneumonia (41%). These were based on very small numbers in the surveys, with wide confidence intervals. The surveys also seemed to overestimate the decline in malnutrition (20% decline in the statistics), although definitions between severe and moderate malnutrition might differ between the two sources. The decline in the number of accidents was also small in the medical statistics (3%).

### Relationship with child-survival programmes

Causes of death could be classified according to the health programmes targeting them. Four major programmes were selected: the vaccination (EPI) programme targeting measles, whooping cough, tetanus, tuberculosis, diphtheria, and poliomyelitis; case management for diarrhoeal diseases and malnutrition; case management for bacterial infections, such as pneumonia, typhoid, meningitis, septicaemia; and care of the newborn (prematurity, low birth-weight, birth trauma). The results indicate that mortality from vaccine-preventable diseases underwent the largest decline (-87%), followed by case management of diarrhoea and malnutrition (-52%) and of other infections (-47%), all faster than the average decline. In contrast, other causes lumped together changed little, and the differences were only borderline significant (Table 5). This reveals the major success of selective primary healthcare programmes and the gaps to be bridged for ensuring further advances, particularly for care of newborns and for ALRIs.

**Table 5:** Mortality change by main categories of causes of death, Morocco, 1987-1996

Main disease category	Mortality level (per 1,000)		Mortality change	
	ECCD-1	ECCD-2	RR	p value
Target of main health interventions				
Vaccine-preventable diseases	11.71	1.37	0.117	0.000∗
Diarrhoea and malnutrition	25.61	10.70	0.418	0.000∗
Other infectious diseases	24.52	11.52	0.470	0.000∗
Other categories and unknown	42.23	35.80	0.848	0.071
Total	104.1	59.4	0.571	0.000∗

∗Significant p<0.05; ECCD=Enquête sur les Causes et Circonstances de Décès; RR=Relative risk

### Contributions to mortality decline

The net contribution of major health programmes to the decline in mortality could be evaluated by computing the mortality fraction attributable to target diseases. The vaccine-preventable diseases contributed to 23% of the total decline in mortality, diarrhoea, and malnutrition to 33%, and treatment of other infectious diseases to 29%, and the reminder explaining only a small fraction (15%).

## DISCUSSION

Morocco seems to be the first country to have attempted using verbal autopsies at a national level to estimate cause-specific death rates and to have repeated the experiment nine years later for measuring the changes and evaluating the impact of interventions. Since then, other countries have made similar attempts, although with only limited experience so far.

Assessing the causes of death is a serious advance over documenting the plain mortality levels for all causes combined and trends for understanding the determinants of child mortality and the impact of health programmes. In the case of Morocco, the major success of the vaccination programme became obvious and other advances made in the prevention and treatment of diarrhoea and severe malnutrition. Furthermore, the surveys showed that the ALRI programme was lagging behind and could provide a measure of the potential gains in child survival if the number of ALRI-related deaths could be reduced.

There are obviously limitations to using verbal autopsies at a national level. A first limitation is the sample size. By selecting deaths recorded by other demographic surveys, the samples obtained were small, and some causes of death were represented only by a few cases, which produced wide confidence intervals for cause-specific death rates. However, the pattern remained robust for the leading causes and confirmed by the comparison with statistics on medical causes of death.

A second limitation of verbal autopsies is the quality of diagnosis and the sensitivity and specificity compared to medical diagnoses. If some causes seemed well-recorded, others were more problematic, particularly typhoid, meningitis, and septicaemia. These causes require more study, and it might be possible to improve the questionnaires to better tackle them. However, a proper assessment also requires that they could be compared with a gold standard, whereas proper coding of causes of death for these diseases is still problematic in hospitals.

A striking result of the first ECCD survey was the high mortality from neonatal tetanus, even in cities, and from diarrhoeal diseases. Obviously, these problems were resolved by the time of the second survey and by itself the related interventions (vaccination of pregnant women and hygiene at delivery/oral rehydration and child feeding) made a large contribution to the decline in mortality. Furthermore, it seems that neonatal tetanus has been eliminated by 2002 in Morocco (25), which shows that simple interventions could have a marked effect on child survival in this type of situation.

Morocco underwent a dramatic decline in mortality over the period studied, rapid by any standard, and impressive given the relatively low level of income in the country (about US$ 2,500 in parity purchasing power), the slow economic growth (<1% a year), the lag in female education, and the geographical isolation of large sections of the country. These mortality trends appeared not only as the continuation of the previous trends, but also as the effect of concerted actions revealed by the changing rates of cause-specific mortality. This does not mean that long-term improvements in other factors did not play a role, but that child-survival programmes had a significant additional impact. Vaccine-preventable diseases would not have declined so rapidly without the strong EPI, and, likewise, for diarrhoea and malnutrition, and for case management of infectious diseases. Documenting the impact of these programmes is important, as a reward for the health personnel who implemented them, and as an incentive for ministries and donor agencies to continue the fight against diseases of young children.
